# Research Progress in Bat Dietary Analysis: Methods, Applications, and Future Perspectives

**DOI:** 10.3390/biology15060449

**Published:** 2026-03-10

**Authors:** Qiulin Guo, Yingying Liu, Sen Liu, Yang Geng

**Affiliations:** 1 College of Life Science, Jilin Agricultural University, 2888 Xincheng Street, Changchun 130118, China; 2 Jilin Provincial Key Laboratory of Animal Resource Conservation and Utilization, Northeast Normal University, 2555 Jingyue Street, Changchun 130117, China; 3 College of Life Sciences, Henan Normal University, Xinxiang 453007, China

**Keywords:** Chiroptera, DNA metabarcoding, stable isotopes, ecosystem services, trophic ecology, pest control, molecular diet analysis, conservation

## Abstract

Bats (Chiroptera) provide critical ecosystem services, including pest suppression, pollination, and seed dispersal. Understanding their dietary ecology is essential for conservation management yet has historically been constrained by methodological limitations. This review synthesizes advances in bat dietary analysis over the past several decades, from traditional morphological and stable isotope approaches to the revolutionary DNA metabarcoding techniques that now dominate the field. We systematically evaluate the strengths and limitations of each methodological approach and examine how molecular methods have transformed our understanding of bat trophic ecology. Research progress across major feeding guilds—insectivorous, frugivorous, nectarivorous, carnivorous, and sanguivorous bats—is examined, with emphasis on recent discoveries enabled by molecular techniques. We discuss ecological and conservation applications, including ecosystem service quantification, food web construction, and responses to environmental change. Finally, we identify priority directions for future research, including long-read sequencing technologies, multi-method integration, reference database expansion, and One Health applications. This synthesis provides guidance for researchers selecting appropriate analytical approaches and highlights the critical role of dietary studies in bat conservation amid accelerating global change.

## Simple Summary

Bats play a keystone role in ecosystems, providing quantifiable benefits through arthropod suppression, pollination, and seed dispersal. Reliable dietary inference is therefore essential for ecosystem-service assessment and evidence-based conservation. However, dietary characterization remains challenging because bats forage at night, feed cryptically, and digest rapidly. In this review, we synthesize progress in bat dietary analysis over the past several decades, spanning traditional microscopic identification of prey remains, stable isotope approaches that integrate assimilated resources over time, and DNA metabarcoding, which can detect prey and plant resources from fecal samples with much higher taxonomic resolution. We summarize advances across major feeding guilds (insectivorous, frugivorous, nectarivorous, carnivorous, and sanguivorous bats) and show how molecular diets have revealed unexpected temporal and spatial variation. These data are increasingly used to quantify ecosystem services, construct trophic interaction networks, and evaluate responses to habitat fragmentation, agricultural intensification, urbanization, and climate-driven shifts in resource phenology. We also outline priorities for the next phase of research, including long-read sequencing, multi-method integration, standardized protocols, and One Health applications linking dietary surveillance with disease ecology and risk management.

## 1. Introduction

Bats (Chiroptera) represent the second most diverse mammalian order after rodents, comprising over 1470 described species distributed across nearly all terrestrial ecosystems except polar regions and remote oceanic islands [1,2]. This remarkable diversity, accounting for approximately 20% of mammalian species, reflects successful exploitation of extraordinarily varied dietary niches over more than 50 million years of evolutionary history [3]. Bats exhibit unparalleled dietary diversity among mammals, consuming resources ranging from insects and other arthropods to fruits, nectar, pollen, vertebrates, blood, and fish [4]. This trophic flexibility has positioned bats as key components of terrestrial food webs and critical providers of ecosystem services across tropical and temperate ecosystems worldwide [5,6].

The ecological and economic significance of bats has gained increasing recognition as research quantifies their contributions to ecosystem function [7]. Insectivorous bats, comprising approximately 70% of chiropteran diversity, provide invaluable pest suppression services in agricultural and urban landscapes [8,9]. Colonies of Brazilian free-tailed bats (*Tadarida brasiliensis*) consume several tons of insects nightly, including major agricultural pests such as cotton bollworm moths (*Helicoverpa zea*). In south-central Texas, the pest control service provided by these bats for cotton production is valued at approximately $741,000 annually [10]. On a broader scale, insectivorous bats are estimated to provide pest suppression services worth over $1 billion globally for corn crops alone [11]. Beyond pest control, frugivorous and nectarivorous bats serve as essential mutualistic partners in plant reproduction. Phyllostomid bats in the Neotropics and pteropodid bats in the Old World tropics pollinate and disperse seeds for hundreds of plant species, including economically important crops such as durian, agave, and various tropical fruits [12,13]. Bat-mediated seed dispersal is particularly crucial for forest regeneration, as frugivorous species preferentially forage in disturbed habitats and deposit seeds in open areas, facilitating successional processes essential for tropical forest recovery [14,15].

Accurate characterization of bat diets is fundamental to understanding ecological roles, trophic interactions, and conservation requirements [16,17]. Dietary information informs ecosystem service assessments, enables identification of critical foraging habitats, and facilitates prediction of responses to environmental change. However, the nocturnal and volant nature of bats presents considerable methodological challenges. Traditional approaches relying on morphological identification of prey remains have provided valuable baseline data but suffer from well-documented limitations including low taxonomic resolution, observer bias, and inability to detect soft-bodied prey [18,19]. The advent of molecular techniques, particularly DNA metabarcoding, has revolutionized bat dietary analysis by enabling high-throughput, species-level identification from non-invasively collected fecal samples [20,21].

This review synthesizes methodological advances and ecological insights in bat dietary analysis. We examine traditional morphological and isotopic approaches alongside molecular methods, summarize research progress across feeding guilds, discuss conservation applications, and identify future research priorities. By integrating methodological perspectives with ecological applications, this synthesis guides researchers in selecting appropriate analytical approaches while highlighting the critical importance of dietary studies for bat conservation.

## 2. Dietary Analysis Methods

### 2.1. Traditional Morphological Approaches

Morphological identification represents the earliest and historically most widely employed approach for investigating bat feeding ecology [18,22]. This methodology relies on microscopic examination of fecal pellets or stomach contents to identify prey remains based on diagnostic morphological features [23]. For insectivorous bats, identification relies on recognition of persistent chitinous structures including lepidopteran wing scales, coleopteran elytra and mandibles, dipteran halteres, and hymenopteran stings [24]. For frugivorous species, seeds, fruit pulp fibers, and pollen grains are identified through comparison with botanical reference collections [25].

**Figure 1:**
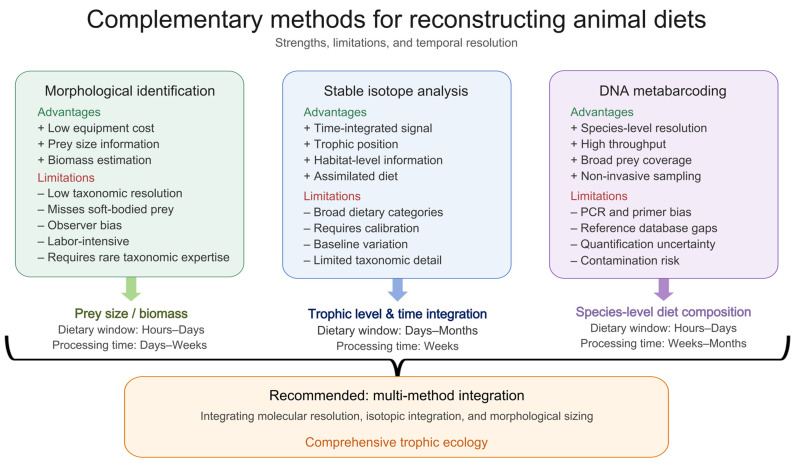
Comparison of bat dietary analysis methods.

Each dietary analysis method presents distinct trade-offs regarding taxonomic resolution, required analytical processing time, and the temporal dietary window captured (Figure 1). Despite methodological advances, morphological analysis offers notable advantages that ensure its continued application. The approach can be implemented with relatively modest equipment and well-curated reference collections, which lowers the barrier in terms of initial capital expenditure and can be feasible for resource-limited programs [26]. Additionally, morphological analysis uniquely provides information on prey size and developmental stage, enabling biomass estimation through regression relationships that molecular methods cannot directly capture [27]. The approach permits distinction between actively captured prey and incidentally ingested items based on fragment completeness and digestion state. Accordingly, morphological analysis is most appropriate when biomass- or prey-size-based dietary inference is required, when hard-bodied prey can be reliably identified, or when laboratory infrastructure for molecular workflows is unavailable.

However, substantial limitations constrain the utility of morphological identification for comprehensive dietary characterization. First, taxonomic resolution varies considerably; many prey are identifiable only to the family or order level due to convergent morphologies among taxa [28]. Second, differential digestibility creates severe detection biases. Soft-bodied prey including spiders, earthworms, and larvae lack persistent structures and are systematically undetectable, creating biases toward hard-bodied arthropods that can substantially misrepresent actual dietary composition [29]. Finally, critical hidden costs and a reliance on specialized expertise significantly limit scalability [19]. While equipment costs are modest, the method is highly labor-intensive and depends on experts to recognize diagnostic features of fragmented remains. Consequently, when personnel time and training are factored in, the total cost per sample can be substantial and may actually exceed that of metabarcoding in large-scale studies. Whereas metabarcoding requires higher upfront laboratory investments, it ultimately offers superior throughput and scalability. Despite these constraints, morphological analysis retains important roles for prey size quantification and validation of molecular detections, with integration rather than replacement representing optimal practice [30].

### 2.2. Stable Isotope Analysis

Stable isotope analysis exploits predictable fractionation of naturally occurring isotopes through food webs, providing temporal integration of dietary information that complements taxonomic methods [31,32]. Carbon isotope ratios (δ^13^C) exhibit relatively conservative trophic transfer and trace primary production sources, enabling distinction between C_3_ and C_4_-based food webs [33]. This baseline variation propagates through consumer communities, differentiating bats foraging in forests dominated by C_3_ vegetation versus agricultural landscapes with C_4_ crops. Nitrogen isotope ratios (δ^15^N), enriched approximately 3–4‰ per trophic level, provide the primary basis for trophic position estimation [34]. Insectivorous bats typically exhibit elevated δ^15^N values indicating secondary or tertiary consumer status, while frugivorous species display lower values reflecting primary consumer positions.

Sulfur isotope ratios (δ^34^S) provide complementary information on habitat use patterns [35]. Unlike carbon and nitrogen, sulfur isotopes exhibit minimal trophic fractionation but display substantial spatial variation associated with marine influences and geological substrates, making δ^34^S valuable for identifying marine-derived prey consumption by coastal bat populations. The integration of multiple isotope systems within Bayesian mixing models enables quantitative estimation of proportional contributions from different food sources, while isotopic niche metrics characterize dietary breadth and interspecific overlap [36].

A critical consideration involves tissue selection, as different tissues integrate dietary information over distinct timescales determined by metabolic turnover rates [37]. Blood plasma typically integrates diet over short timescales (often on the order of days) [38], while metabolically inert tissues including fur and claws preserve signatures from synthesis periods potentially months prior to sampling [39]. This temporal hierarchy enables retrospective dietary reconstruction when tissue-specific discrimination factors are adequately characterized.

Key limitations include broad taxonomic resolution distinguishing dietary categories rather than species, tissue-specific discrimination factors requiring species-specific calibration, and potential confounding by nutritional stress [39]. Nevertheless, stable isotope analysis provides irreplaceable temporal integration and trophic context that complement the taxonomic precision of molecular methods, making multi-method approaches incorporating both techniques increasingly standard practice [40].

### 2.3. DNA Barcoding and Metabarcoding

The development of DNA-based dietary analysis has fundamentally transformed bat trophic ecology research over the past fifteen years [20,41]. DNA barcoding employs standardized genetic markers for species identification, with the mitochondrial cytochrome c oxidase subunit I (*COI*) gene serving as the primary animal barcode [42]. Plant identification employs chloroplast markers ribulose bisphosphate carboxylase large (*rbcL*) and megakaryocyte-associated tyrosine kinase (*matK*) alongside nuclear ribosomal internal transcribed spacer 2 (ITS2) regions [43,44]. Early dietary applications used Sanger sequencing of individual prey items, but this approach cannot resolve the mixed templates characteristic of dietary samples containing multiple prey species [45].

DNA metabarcoding revolutionized dietary analysis by combining barcoding principles with high-throughput sequencing (HTS) platforms, enabling simultaneous identification of multiple prey taxa from complex fecal samples [21]. The massively parallel sequencing capacity of HTS platforms generates millions of sequences per run, providing sufficient depth to detect both abundant and rare dietary components while simultaneously processing hundreds of samples. This transformative capability has made molecular dietary analysis the contemporary standard for comprehensive characterization of bat trophic ecology.

The metabarcoding workflow encompasses interconnected steps requiring careful optimization. Sample collection employs non-invasive fecal sampling, with pellets preserved in 95–100% ethanol or dried with silica gel, then stored at reduced temperatures to minimize DNA degradation [46]. DNA extraction utilizes commercial kits optimized for fecal matrices incorporating inhibitor removal, with protocol modifications including extended lysis and bead-beating enhancing recovery from recalcitrant prey structures [46,47]. Primer selection represents perhaps the most consequential methodological decision, determining taxonomic scope and resolution. For arthropod prey, *COI* primers including the widely adopted Zeale primers targeting ~157 bp fragments dominate insectivorous bat studies [48]. However, their taxonomic coverage is not uniform, and reduced amplification efficiency has been reported for some Diptera and Hemiptera; therefore, in silico evaluation and complementary primer sets are recommended when broad arthropod coverage is required [30,49].

In addition, plant dietary components require alternative markers: the *trnL* P6 loop performs well with degraded DNA but often provides coarser taxonomic resolution, whereas ITS2 may improve species-level discrimination but can amplify less consistently; *rbcL* offers broad universality but similarly trades off resolution [43,44,50]. Omnivorous diets require multi-marker approaches, typically pairing an arthropod *COI* mini-barcode with plant markers [48,50]. Multi-marker assays can be implemented as separate PCRs (recommended in most cases) or multiplex PCRs (useful when DNA quantity is limited). Separate PCRs allow marker-specific optimization and reduce inter-primer competition, improving reproducibility, whereas multiplexing reduces cost and sample use but may increase preferential amplification and uneven marker recovery [51]. When integrating results, marker-specific filtering and cautious cross-marker comparison are essential, and read abundances should not be treated as directly comparable across markers [52].

Given the sensitivity of high-throughput sequencing to contamination and amplification bias, rigorous quality control is essential throughout the workflow. We recommend including extraction blanks and PCR negative controls to detect laboratory and reagent contamination, as well as positive controls to benchmark amplification success, sequencing performance, and bioinformatic accuracy. Technical replicates can further improve reliability, and filtering criteria should be reported transparently, ideally informed by control profiles [17,51].

Sequencing platforms have diversified considerably. Illumina short-read sequencing (MiSeq, NovaSeq) currently dominates due to high throughput, low error rates, and established bioinformatic infrastructure [53]. Bioinformatic processing through pipelines including QIIME2, DADA2, and OBITools performs quality filtering, sequence clustering or denoising generating operational taxonomic units (OTUs) or amplicon sequence variants (ASVs), and taxonomic assignment against reference databases [54,55,56]. Post-assignment filtering removes likely contaminants and artifacts based on negative control profiles and minimum abundance thresholds [57].

### 2.4. Technical Challenges and Biases

Despite transformative capabilities, metabarcoding is subject to biases requiring careful consideration in study design and interpretation [52]. DNA degradation during digestive processing progressively fragments prey DNA, favoring detection of recently consumed or digestion-resistant items [58]. Differential digestibility creates systematic biases, with hard-bodied arthropods possessing protective exoskeletons retaining identifiable DNA longer than soft-bodied prey [46,58,59]. PCR amplification biases arise from variation in primer–template binding efficiency, causing differential amplification that distorts relationships between read abundance and actual consumption [60]. In bat dietary metabarcoding, the co-amplification of host DNA often dominates sequencing libraries, limiting prey detection. To mitigate this, mammal-blocking primers are widely used to suppress bat DNA during PCR. However, their efficiency varies, and incomplete blocking persists. Moreover, these primers carry the inherent risk of inadvertently suppressing closely related prey taxa. Therefore, blocking primers require careful validation to balance effective host suppression with unbiased prey detection [49,52].

Reference database limitations fundamentally constrain taxonomic assignment accuracy. Sequences lacking close database matches can only be assigned to higher taxonomic levels, with substantial gaps persisting for tropical arthropods and plants despite ongoing barcoding initiatives [61]. The relationship between sequence read abundance and dietary biomass is distorted by multiple factors including differential DNA content among prey, extraction efficiency variation, and amplification biases, limiting quantitative inference from standard metabarcoding [62]. To navigate these biases, researchers should adopt a structured decision framework. For general ecological profiling, researchers should default to frequency of occurrence metrics, reporting the proportion of samples containing each dietary item, which provide more reliable, though less informative, dietary summaries than abundance-based estimates [63]. However, when true abundance estimation is strictly required, experimental designs must integrate recent methodological advances. Controlled mock communities and feeding experiments have been used to derive taxon-specific correction factors that partially mitigate amplification bias. Spike-in approaches incorporating synthetic internal standards during extraction or PCR allow calibration of sequencing output across samples, and independent validation using qPCR or droplet digital PCR can further refine biomass inference. However, quantitative metabarcoding remains context-dependent, and substantial debate persists regarding the reliability of read abundance as a proxy for dietary biomass [52,62]. Ongoing discussions emphasize that calibration experiments and transparent reporting standards are essential for robust ecological interpretation.

## 3. Dietary Research Across Feeding Guilds

### 3.1. Insectivorous Bats

**Figure 2:**
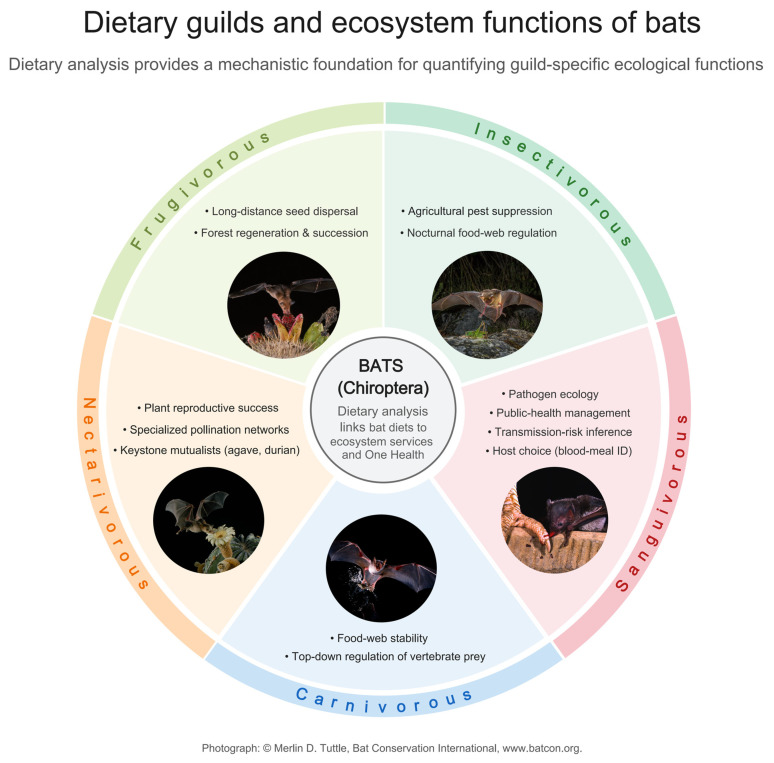
Dietary guilds as mechanistic pathways linking bats to ecosystem functions.

Insectivorous bats, comprising approximately 70% of chiropteran diversity, have received extensive dietary research attention driven by their pest suppression services and relative methodological accessibility [8,64]. Metabarcoding has documented consumption of major agricultural pests with species-level precision unattainable through morphological methods (Figure 2), enabling robust ecosystem service quantification [65,66].

North American studies have documented substantial pest consumption by colonial species. Brazilian free-tailed bats consume cotton bollworm, corn earworm, and tobacco budworm moths, with economic models estimating annual pest control values in south-central Texas alone at hundreds of millions of dollars [10,67]. European research documents consumption of vineyard pests (*Lobesia botrana*, *Eupoecilia ambiguella*), corn pests (*Spodoptera frugiperda*), and olive pests (*Prays oleae*) by *Rhinolophus* and *Myotis* species, supporting conservation biological control strategies [68,69]. Asian studies reveal predation on rice pests including planthoppers and stem borers by horseshoe bats and leaf-nosed bats, contributing to food security across the region [70]. African research highlights the consumption of major macadamia pests by members of the Molossidae and Vespertilionidae, with empirical models demonstrating significant avoided economic damage in South African orchards [71]. Southeast Asian studies in tropical agroforestry demonstrate that insectivorous bats contribute significantly to cacao yield through suppression of herbivorous insects, underscoring their indispensability for tropical agricultural sustainability and local livelihoods [72].

Molecular dietary analysis has revealed pronounced temporal and spatial dietary variation previously invisible to morphological methods. Seasonal shifts track arthropod phenology, with spring diets emphasizing early-emerging Diptera and Lepidoptera, transitioning through peak summer diversity, to autumn emphasis on late-season prey [73]. Habitat-specific analyses document distinct prey assemblages in agricultural versus forested landscapes, with agricultural areas supporting pest-dominated diets [74]. Resource partitioning among sympatric species, enabling coexistence of species-rich assemblages, emerges clearly from molecular data revealing fine-scale dietary differentiation [75]. Despite these advances, synthesizing insectivorous dietary research reveals significant methodological inconsistencies. Molecular studies frequently report “hyper-diverse” diets compared to traditional morphological analyses. However, this hyper-diversity may partially reflect artifacts such as secondary predation (detecting the prey of the prey) or extreme sensitivity to trace environmental DNA, which can artificially inflate niche breadth estimates [47,52]. Furthermore, a pronounced geographical knowledge gap limits global synthesis: while temperate agricultural systems are well-characterized, tropical insectivorous food webs remain poorly resolved due to incomplete arthropod reference databases [62]. To navigate these complexities, selecting the optimal analytical method depends strictly on the specific research question. While DNA metabarcoding is indispensable for resolving the fine-scale taxonomy of soft-bodied agricultural pests, morphological analysis remains essential for estimating hard-bodied prey biomass, and stable isotopes are required for integrating long-term trophic shifts. Ultimately, researchers must adopt a question-driven decision framework rather than relying on a single universal approach [40,47].

### 3.2. Frugivorous Bats

Frugivorous bats, concentrated in the Neotropical Phyllostomidae and Old World Pteropodidae, serve as critical seed dispersers maintaining tropical forest diversity and facilitating regeneration on degraded lands [14,76]. Metabarcoding has revealed plant-bat interaction networks far more complex than morphological studies suggested, with individual bat species consuming fruits from dozens of plant species across families [77,78].

Pioneer plant species bearing small-seeded fruits are particularly dependent on bat dispersal. *Cecropia*, *Piper*, *Solanum*, and *Ficus* constitute core dietary components whose seeds pass intact through bat digestive systems, deposited in feces during subsequent foraging or at roost sites [79]. Network analyses characterize the modularity, nestedness, and robustness of seed dispersal interactions, identifying keystone bat species whose loss would disproportionately impact dispersal function [80]. The long-distance flight capabilities of bats enable seed movement across fragmented landscapes, connecting isolated forest patches and facilitating gene flow among plant populations critical for regeneration in anthropogenically modified landscapes [81].

Molecular dietary data have challenged traditional characterizations of frugivores as dietary specialists, revealing flexibility responding to temporal and spatial fruit availability variation [82]. At the same time, evidence for genuine specialization persists. Fig specialists among both Neotropical and Old World frugivores consume *Ficus* disproportionately relative to availability, potentially reflecting sensory adaptations for fig detection [83]. Nevertheless, a critical guild-specific methodological challenge remains in distinguishing core nutritional dependence from incidental ingestion. Current metabarcoding approaches struggle to differentiate between seeds that are physically dispersed (swallowed and passed intact) versus fruits where only pulp is consumed and seeds are expelled at the feeding roost [16,20]. Consequently, to resolve these methodological contradictions, future studies must transition toward multi-method integration, combining high-resolution molecular networks with quantitative stable isotope mixing models to confirm whether the vast array of detected plants meaningfully contributes to assimilated biomass [40,84].

### 3.3. Nectarivorous Bats

Nectarivorous bats provide pollination services for hundreds of plant species including economically important crops [12,85]. Pollen metabarcoding from fur and feces documents pollination interactions that observational methods cannot detect, capturing cumulative pollen loads from multiple flower visits during nocturnal foraging [86].

Wild plant pollination involves diverse families exhibiting chiropterophilous floral syndromes—nocturnal anthesis, pale coloration, robust flowers, and copious nectar. Columnar cacti in arid Americas depend almost exclusively on bat pollination, with glossophagine bats transporting pollen among widely dispersed plants [87]. Crop pollination by bats includes agave (supporting multi-billion-dollar tequila and mezcal industries), durian (among Southeast Asia’s most valuable fruits), and various tropical species, with molecular dietary data supporting economic service valuations influencing agricultural and conservation policy [88].

Network analyses reveal a nested pollination structure conferring robustness against species loss [89]. Migratory nectarivores including *Leptonycteris* species track flowering phenology along elevational and latitudinal gradients, providing pollination services across landscapes no resident species could supply and highlighting vulnerability to climate-driven phenological mismatches [90,91]. However, a critical interpretive challenge remains: pollen metabarcoding may capture environmental contamination rather than legitimate pollination visits [92]. Therefore, molecular presence does not inherently confirm functional pollination. To overcome this limitation and accurately map these mutualisms, the field must increasingly integrate molecular dietary networks with empirical camera-trapping data and long-term phenological monitoring [13,85].

### 3.4. Carnivorous and Sanguivorous Bats

Carnivorous bats consuming vertebrate prey and sanguivorous vampire bats present unique dietary analysis challenges that molecular methods address more effectively than any traditional approach. Vertebrate tissues lack the diagnostic hard structures enabling morphological identification, making DNA-based methods essential for prey characterization [93].

The spectral bat (*Vampyrum spectrum*), the largest New World bat, consumes birds, rodents, and other bats, functioning as an apex predator in Neotropical ecosystems [94]. Fringe-lipped bats (*Trachops cirrhosus*) selectively prey on calling frogs, with metabarcoding revealing selective predation patterns [95]. Piscivorous species including *Noctilio leporinus* consume diverse fish alongside aquatic invertebrates, with molecular identification revealing more catholic diets than specialized piscivory implies [96].

Vampire bats (Desmodontinae) require blood meal identification for understanding host selection and disease transmission dynamics [97]. Common vampire bats (*Desmodus rotundus*) exhibit flexible host selection, predominantly feeding on domestic livestock in agricultural regions but exploiting wildlife including peccaries, deer, and tapirs in forested areas [31,98]. Similarly, human blood consumption has been molecularly documented in the hairy-legged vampire bat (*Diphylla ecaudata*) in northeastern Brazil, indicating dietary flexibility when preferred hosts become scarce, with significant public health implications [99].

Blood meal identification directly informs rabies epidemiology by documenting which host populations experience feeding pressure and thus transmission risk [100]. Integration of dietary surveillance with wildlife disease monitoring enhances early warning capacity and guides targeted management interventions balancing public health, agricultural, and conservation concerns [101]. However, synthesizing these findings highlights unique methodological inconsistencies. The close phylogenetic relationship between carnivorous bats and their mammalian or avian prey necessitates the use of host-blocking primers, which carry the inherent risk of inadvertently suppressing closely related prey DNA and skewing dietary profiles [49]. Overcoming these biases via customized assays or CRISPR-based depletion is essential for accurately resolving cryptic trophic interactions and long-term dietary plasticity [102].

## 4. Ecological and Conservation Applications

### 4.1. Food Web Construction and Trophic Ecology

Molecular dietary data enable construction of quantitative food webs mapping bat-prey interactions with unprecedented resolution [103]. Species-level identification permits calculation of network metrics including connectance, linkage density, and interaction evenness characterizing food web structure [104]. Quantifying these network metrics allows ecologists to identify “keystone prey”—specific arthropod or plant species that disproportionately sustain bat communities during resource bottlenecks. Furthermore, by comparing network robustness across different disturbance regimes, researchers can transition from merely descriptive food webs to predictive models that forecast how bat communities might collapse or reorganize under future biodiversity loss scenarios. Bipartite network representations reveal dietary guilds, distinguish generalist from specialist consumers, and identify structural properties conferring network robustness.

Niche partitioning analyses test coexistence mechanisms among sympatric species through quantification of dietary overlap [105]. Molecular data have documented significant dietary differentiation among morphologically similar species foraging in shared habitats, suggesting fine-scale resource partitioning invisible to traditional methods [74]. The combination of metabarcoding for taxonomic resolution with stable isotopes for temporal integration and trophic context provides the most comprehensive current framework for characterizing bat trophic ecology [40].

### 4.2. Responses to Environmental Change

Dietary analysis provides sensitive indicators of environmental change impacts that may precede demographic consequences [106]. Forest fragmentation significantly alters prey communities and subsequent consumption patterns. For example, Tobisch et al. (2025) applied DNA metabarcoding to bat diets and detected 405 arthropod species, further demonstrating that greater forest and grassland cover within a 2 km foraging buffer was significantly and positively associated with dietary species richness, while high fragmentation restricts access to diverse prey [107]. Similarly, agricultural intensification dramatically reduces prey availability and alters foraging efficiency; recent large-scale assessments have quantified that bat foraging activity and social calls are up to four times higher in structurally diverse, low-intensity agricultural habitats than in intensive conventional croplands, consistent with severe depletion of insect resources under intensified management [108].

Climate change affects arthropod phenology, abundance, and distribution in ways cascading to bat consumers [109]. Phenological shifts in insect emergence may create temporal mismatches with bat activity, potentially reducing prey availability during critical foraging periods. Urban bat populations exhibit dietary adaptations including exploitation of light-attracted insects and synanthropic prey, with some species thriving while others decline in urban environments [110].

### 4.3. Conservation Management Applications

Dietary data inform multiple conservation applications essential for effective bat protection [9]. Critical habitat identification extends beyond roost protection to encompass foraging areas supporting essential prey resources [111]. Documenting prey consumption enables mapping of foraging habitats complementing roost-focused conservation, with landscape-scale planning incorporating both requirements.

Ecosystem service valuations based on species-level dietary data provide economic arguments resonating with policymakers [7]. Quantification of pest suppression, pollination, and seed dispersal services depends on accurate dietary documentation, with valuations reaching billions of dollars annually supporting conservation investment [11]. Restoration planning informed by prey requirements enables habitat enhancements delivering foraging benefits, while dietary monitoring tracks functional recovery that vegetation assessments alone cannot capture [112].

Furthermore, dietary data are increasingly utilized to design targeted agri-environment schemes [64,108]. For instance, identifying the exact prey base of insectivorous bats allows land managers to retain specific non-crop vegetation—such as hedgerows or riparian buffers—that supports bat foraging during non-outbreak periods. In tropical forest restoration, molecular identification of core dietary plants consumed by frugivorous bats enables practitioners to proactively cultivate these “magnet species” [77,79]. Planting these specific pioneer plants attracts bat visitation, thereby accelerating natural successional processes and enhancing structural connectivity across fragmented landscapes [14].

For threatened species, dietary indicators may detect population stress before demographic consequences become apparent [113]. Long-term monitoring incorporating dietary assessment alongside population estimation provides comprehensive evaluation of conservation status, with declining dietary breadth or quality signaling threats operating through foraging ecology.

## 5. Future Directions

### 5.1. Methodological Advances

**Figure 3:**
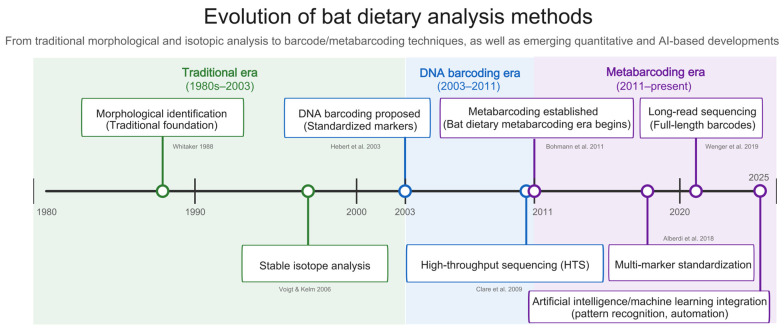
Evolution of bat dietary analysis methods [17,18,21,31,42,45,114].

The field of bat dietary analysis has undergone remarkable transformation over the past four decades, evolving through distinct methodological eras (Figure 3). The traditional era (1980s–2003) established foundational approaches through morphological identification and stable isotope analysis. The DNA barcoding era (2003–2011) began with Hebert et al.’s (2003) proposal of standardized genetic markers [42], followed by Clare et al.’s (2009) early molecular prey-detection applications in bat diet studies [45]. The metabarcoding era (2011–present), catalyzed by early HTS applications in bat dietary research, has undergone rapid refinement—including multi-marker standardization and long-read sequencing applications—and has coincided with a rapid expansion of bat dietary studies, reflecting the accessibility and scalability of HTS-based workflows.

Building upon this foundation, emerging technologies promise continued advancement. Long-read platforms including Pacific Biosciences HiFi and Oxford Nanopore Technologies (ONT) are poised to resolve many limitations of short-read platforms. A primary advantage of long-read sequencing is the ability to span entire barcode regions, which dramatically improves taxonomic resolution to the species level and reduces PCR-induced chimera formation [115]. However, critical limitations currently constrain their widespread adoption; ONT platforms still grapple with higher raw sequencing error rates that necessitate highly specialized, error-correcting bioinformatic pipelines [116], while PacBio HiFi—despite its exceptional accuracy via circular consensus sequencing—remains limited by higher per-sample costs and lower multiplexing scalability compared to established short-read platforms. Furthermore, portable nanopore devices offer field-deployable analysis for remote biodiversity hotspots where centralized facilities are inaccessible [117]. Quantitative approaches including spike-in standards and digital PCR may improve abundance estimation accuracy, addressing persistent quantification limitations [118]. The integration of artificial intelligence and machine learning represents an emerging frontier that may further transform dietary data analysis in coming years, for example by facilitating automated bioinformatic processing and integrating multi-modal environmental data to strengthen biodiversity inference [119]. By leveraging these computational tools, future dietary studies may transition from descriptive summaries toward predictive modeling of complex predator–prey ecological networks.

### 5.2. Multi-Method Integration

Recognition that no single method provides complete dietary characterization drives development of multi-method frameworks leveraging complementary strengths [40]. Combining metabarcoding with stable isotopes integrates taxonomic resolution with temporal integration synergistically. Morphological data fusion provides prey size information molecular methods cannot capture, enabling biomass-weighted dietary estimation [120]. Machine learning applications for dietary pattern recognition offer emerging analytical capabilities for high-dimensional dietary datasets [121].

### 5.3. Global Research Priorities

Geographic bias toward temperate regions limits tropical research where bat diversity peaks but database coverage remains incomplete [122]. Addressing this imbalance requires coordinated structural initiatives rather than incremental case studies. First, targeted regional barcoding initiatives must be funded to establish dedicated regional database nodes, particularly in biodiversity hotspots. Systematically addressing coverage gaps for taxa frequently encountered in bat diets would substantially improve species-level assignment and reduce the current overrepresentation of temperate taxa [123]. Second, equitable collaborative funding models must pair resource-rich institutions with local tropical ecologists to establish these monitoring networks. Protocol standardization enabling cross-study comparison requires community-developed reporting standards and benchmark datasets for inter-laboratory validation. Finally, long-term dietary monitoring programs at sentinel sites would generate temporal baselines for detecting change, with integration into existing bat monitoring networks leveraging established sampling infrastructure [51,124].

### 5.4. One Health Perspectives

Bat dietary ecology intersects disease ecology through multiple pathways with implications for pandemic preparedness [125]. Prey consumption exposes bats to pathogens circulating in prey populations, with dietary generalists potentially encountering diverse pathogen sources [126]. Vampire bat host identification informs rabies management by documenting transmission risk across host communities [100]. Furthermore, tropical case studies uniquely illustrate how environmentally driven dietary shifts exacerbate zoonotic risks. In paleotropical and Australasian regions, rapid deforestation forces frugivorous and nectarivorous flying foxes (*Pteropus*) to shift their dietary reliance from native forests to cultivated orchards and urban landscapes. This dietary displacement significantly increases spatial overlap and contact rates between bats, livestock, and humans, a pathway classically linked to the emergence of the Nipah virus [127]. More recently, large-scale macroecological analyses have demonstrated that acute shortages in native tropical dietary resources directly drive pulses of Hendra virus spillover, underscoring the critical link between tropical bat foraging ecology and global pandemic preparedness [128]. Dietary surveillance could detect pathogen circulation in arthropod vectors through analysis of insectivorous bat feces, supporting integrated One Health monitoring combining ecological and epidemiological objectives [129].

Beyond traditional host identification, dietary metabarcoding of insectivorous species offers a vastly underutilized, non-invasive surveillance tool for vector-borne diseases [68]. By detecting disease vectors—such as mosquitoes, ticks, or phlebotomine sand flies—within bat feces, researchers can continuously monitor the spatiotemporal distribution of these vectors without relying on labor-intensive trapping. Future initiatives should aim to integrate this dietary surveillance directly with environmental pathogen screening, simultaneously extracting diet and pathogen data from the same fecal samples to create a robust early-warning system for zoonotic spillover risk [125,129].

## 6. Conclusions

Bat dietary analysis has undergone remarkable transformation through molecular method development, with DNA metabarcoding now enabling species-level prey identification at unprecedented throughput. This methodological revolution has revealed dietary complexity invisible to traditional approaches, documented ecosystem services worth billions of dollars, and provided tools for detecting responses to environmental change.

However, challenges persist. Amplification biases, database gaps particularly in tropical regions, and quantification limitations require careful interpretation of molecular data. In this review, we propose a comprehensive framework that links dietary approaches with ecological issues and conservation, multi-method integration—combining molecular, isotopic, and morphological approaches—represents current best practice for comprehensive dietary characterization. Future advances will emerge from long-read sequencing technologies, expanded reference databases, standardized protocols enabling global synthesis, and integration with One Health frameworks addressing disease dynamics.

As environmental pressures on bat populations intensify through habitat loss, climate change, and emerging diseases, dietary analysis provides critical tools for understanding responses and guiding conservation action. The continued refinement of analytical methods and their application across bat diversity will remain central to chiropteran research and conservation in the coming decades.

## Data Availability

No new data were created or analyzed in this study. Data sharing is not applicable to this article.
